# The association between intimate partner violence, alcohol and depression in family practice

**DOI:** 10.1186/1471-2296-11-72

**Published:** 2010-09-27

**Authors:** Gail Gilchrist, Kelsey Hegarty, Patty Chondros, Helen Herrman, Jane Gunn

**Affiliations:** 1 Substance Use Disorders Research Group, Neuropsychopharmacology Programme; Institut Municipal d'Investigació Mèdica (IMIM)-Hospital del Mar, Barcelona, Spain; 2 Primary Care Research Unit, Department of General Practice, The University of Melbourne, Melbourne, Australia; 3 Orygen Youth Health Research Centre, The University of Melbourne, Melbourne, Australia

## Abstract

**Background:**

Depressive symptoms, intimate partner violence and hazardous drinking are common among patients attending general practice. Despite the high prevalence of these three problems; the relationship between them remains relatively unexplored.

**Methods:**

This paper explores the association between depressive symptoms, ever being afraid of a partner and hazardous drinking using cross-sectional screening data from 7667 randomly selected patients from a large primary care cohort study of 30 metropolitan and rural general practices in Victoria, Australia. The screening postal survey included the Center for Epidemiological Studies Depression Scale, the Fast Alcohol Screening Test and a screening question from the Composite Abuse Scale on ever being afraid of any intimate partner.

**Results:**

23.9% met criteria for depressive symptoms. A higher proportion of females than males (20.8% vs. 7.6%) reported ever being afraid of a partner during their lifetime (OR 3.2, 95%CI 2.5 to 4.0) and a lower proportion of females (12%) than males (25%) were hazardous drinkers (OR 0.4; 95%CI 0.4 to 0.5); and a higher proportion of females than males (20.8% vs. 7.6%) reported ever being afraid of a partner during their lifetime (OR 3.2, 95%CI 2.5 to 4.0). Men and women who had ever been afraid of a partner or who were hazardous drinkers had on average higher depressive symptom scores than those who had never been afraid or who were not hazardous drinkers. There was a stronger association between depressive symptoms and ever been afraid of a partner compared to hazardous drinking for both males (ever afraid of partner; Diff 6.87; 95% CI 5.42, 8.33; p < 0.001 vs. hazardous drinking in last year; Diff 1.07, 95% CI 0.21, 1.94; p = 0.015) and females (ever afraid of partner; Diff 5.26; 95% CI 4.55, 5.97; p < 0.001 vs. hazardous drinking in last year; Diff 2.23, 95% CI 1.35, 3.11; p < 0.001), even after adjusting for age group, income, employment status, marital status, living alone and education level.

**Conclusions:**

Strategies to assist primary care doctors to recognise and manage intimate partner violence and hazardous drinking in patients with depression may lead to better outcomes from management of depression in primary care.

## Background

Depressive symptoms,[1] hazardous drinking [2] and intimate partner violence (IPV) [3] are common among patients attending general practice. In a recent study of primary care patients in six countries, 24-55% met criteria for current depressive symptoms [4]. A recent Australian report estimated that after adjusting for age-sex attendance patterns, 29% of the Australian general practice population was consuming alcohol at a level placing them *"at-risk"*, with a greater proportion of males (36%) at risk than females (24%) [5]. Around a quarter of female general practice patients in Australia have experienced some type of abuse in an adult intimate relationship, with up to 30% having ever been afraid of their partner [3,6]. The prevalence of IPV among male general practice patients has not been documented. Although these problems frequently coexist, the relationship has not been fully explored.

Alcohol and IPV are relatively independent risk factors for depression. Both men and women with a history of depression and men with a history of alcohol dependence are more likely to experience abuse in relationships [7]. IPV may lead to depression among women but not in men; and to alcohol abuse among men but not women [7]. Moreover, hazardous drinking may be a way of coping with or "self-medicating" the negative experience of IPV or depression [8]. A feminist perspective, adopted in the current study, supports the possibility that depression and hazardous drinking are consequences of IPV for women, rather than risk factors for women becoming victims [9,10].

General practitioners (GPs) often lack confidence in detecting and managing hazardous drinking and IPV, particularly the latter [3,11-15]. However, research demonstrates that systematically screening general practice patients for alcohol use and providing brief interventions is cost effective and reduces alcohol consumption [16-18]. Although there is insufficient evidence to support the introduction of routine screening for IPV in general practice,[19] enquiring about fear of a partner/ex-partner is receiving increased attention [20,21] and has significant potential as a stand alone screening item [20].

Hazardous drinking and alcohol disorders are associated with depression,[22] with one study reporting the odds of heavy drinking primary care patients meeting criteria for a major depressive disorder were twice that of non heavy drinking patients [23]. In a US study of primary care patients with probable current depressive disorder, 8% of women and 19% of men reported hazardous drinking [24]. While men consistently report higher levels of alcohol related disorders than women; women with alcohol related disorders consistently report a higher prevalence of depressive disorders than men with alcohol related disorders,[25] potentially related to the higher prevalence of sexual or physical abuse experienced by women [26,27]. Patients with alcohol problems [22,28,29] report poorer outcomes from depression. Higher rates of general practice attendance have been reported among those with alcohol dependence and an additional comorbid psychiatric (mainly affective) disorder compared to those with alcohol dependence alone [29]. Victims of IPV place a great burden on primary care services [30]. Therefore, it is important that GPs identify and address both these psychosocial issues in the course of their depression management, not just alcohol use.

IPV is associated with depression and with alcohol use in several populations. A meta analysis reported the weighted mean prevalence of 44% for depression and 19% for alcohol abuse or dependence among women who reported experiencing IPV in general population and general practice settings [9]. In a cross sectional study of over 1000 consecutive female patients attending general practice in Australia, females who reported IPV were more likely to meet criteria for depressive symptoms (e.g. for severe combined abuse, OR 8.0, 95% CI 4.8 to 13.0) [3]. Among female patients in healthcare settings including primary care, the odds of having experienced IPV in the past year were 2.40 for problem drinking women compared to non-problem drinkers [31]. Furthermore, several studies report an exposure response, with alcohol consumption increasing following IPV [32] to cope with the abuse [8,33,34]. Baseline findings from the current study conducted in Australian general practice highlight that having a substance abuse disorder and reporting ever being afraid of any partner were both associated with persistent depressive symptoms [35].

Despite the high prevalence of these three problems in patients attending general practice; the relationship between depressive symptoms, fear of partner and hazardous drinking remains relatively unexplored. Separate community campaigns focus on each of these major public health problems; however, in clinical practice and education of GPs, IPV has not received the same attention as depression and alcohol related problems [36]. The relationship between depression and hazardous drinking is well understood especially in men; however, the relationship between IPV and depression is less well recognised. While most patients with any or all of these problems are managed in general practice, existing research focuses on patients in psychiatric inpatient settings [1,22] and victims of IPV in shelters [37].

This paper explores the association between depressive symptoms, ever being afraid of a partner and hazardous drinking using cross-sectional data from the *diamond *cohort, a large primary care cohort study underway in Australia [35]. Our objective is to specifically explore if fear of partner (IPV) is as closely associated with depressive symptoms as hazardous drinking. Our hypothesis was that IPV would be more strongly associated with depression than hazardous drinking.

## Methods

Ethics approval was granted by the University of Melbourne's Human Research Ethics Committee (# 030613).

### Design and setting

*diamond *is a prospective longitudinal cohort study of 789 randomly recruited patients with depression from 30 randomly recruited metropolitan and rural general practices in Victoria, Australia [35,38]. This paper reports on data collected at the screening stage of the study using cross sectional data only. Briefly, 17,780 randomly selected patients from 30 general practices who had attended their GP in the previous 12 months for any reason were sent a screening survey between January and December 2005.

### Patient screening survey

The patient screening survey included questions on demographics, lifestyle, health and well-being.

The 20-item Center for Epidemiological Studies Depression Scale (CES-D) measured depressive symptomatology in the previous week [39]. Scores range from 0 to 60 with higher scores indicating more symptoms. A score of ≥ 16 indicates a significant level of depressive symptomatology and identifies a group of patients experiencing major and minor depression and dysthymia (hereafter reported as depressive symptoms). The CES-D has good reliability and validity [39].

The Fast Alcohol Screening Test (FAST) [40] measured hazardous drinking in the past 12 months, i.e. "*a pattern of drinking that is associated with a high risk of psychosocial or physical problems in the future*" [41]. The FAST has four items from which a total score ranging from 0 to 16 is generated. A score of ≥ 3 indicates hazardous drinking in the past 12 months. The four items, originally from the Alcohol Use Disorders Identification Test (AUDIT) [42] cover 1) the frequency of having six (four for women) or more drinks on one occasion; 2) the frequency of being unable to remember what happened the night before because you had been drinking; 3) the frequency of failing to do what was normally expected of you because of drinking; and 4) whether a relative or friend, or a doctor or other health worker had been concerned about your drinking or suggested you cut down. When sensitivity and specificity were tested against the AUDIT,[42] the FAST was reliable across age, sex and location [40]. Good reliability was demonstrated in the inter-correlations between the four items in the FAST (Chronbach's alpha = 0.77) and in the test-retest (Test-retest reliability = 0.81).

Patients were asked whether they had ever been afraid of any intimate partner to determine probable IPV, a question that correlates well with the severe combined abuse dimension of the Composite Abuse Scale (assessment of IPV) among female general practice patients [43]. Asking whether a patient has ever been afraid of any partner has been shown to have good sensitivity and specificity for identifying women who have experienced physical abuse (75.5% sensitivity, 82.4% specificity) and/or severe combined physical and sexual abuse (85.0% sensitivity, 77.7% specificity) in a large sample of women attending family physicians for primary care. It does not perform as well in identifying women who have experienced emotional abuse alone (60.6% sensitivity, 80.4% specificity) [44].

Patients self-rated their overall health as poor/fair/good/excellent, using a question from the Short Form 12 (SF-12),[45] a well validated measure of physical and psychological functioning. Patients were also asked whether they had any long-term illness, health problem or disability that limited their daily activities or the work they could do (including problems related to aging).

### Statistical methods

The outcome measure was CES-D score and was summarised as means and standard deviations (SD) for each of the demographic and psychosocial factors. Confidence intervals and p-values reported were adjusted for the clustering effect of participants nested within general practices. Marginal logistic regression using generalised estimating equations with robust standard errors was used to examine the association between hazardous drinking and sex and probable IPV and sex. Results are reported as odds ratios (OR) with respective 95% confidence intervals (95%CI).

Mixed effects linear regression model using restricted maximum likelihood estimation (REML), treating GP practice as a random effect, was used to examine the association between CES-D score and each factor. The results were reported as differences in mean outcome between individuals with and without the factor, with respective 95%CI and p-values. Interaction was tested between males and females and each of the patient factors. The significance level for testing interaction between factors was set at 10%. When the p-value for interaction was ≥ 10%, the analysis was reported separately by sex. Analysis to explore the association between depressive symptoms, hazardous drinking and ever being afraid of a partner was performed separately by sex.

Multivariable mixed effects linear regression models (REML) were used to test for interaction between hazardous drinking and ever being afraid of partner. Ever afraid of partner and hazardous drinking were fitted in the same model adjusted for all factors with the exception of sexuality: age group, education level, employment status, pension or benefit as main source of income, marital status, lives alone. Sexuality was not adjusted for due to the large proportion of missing data in the sample. Data were analysed using Stata, version 10.1 [46].

## Results

Forty-three percent (7667/17780, 43.1%) of patients returned a screening survey. Patients who returned the survey were on average older (50.9 years (SD, 14.2) vs. 46.2 years (SD, 15.3)) and more likely to be female (66.5% vs. 60.7%) than those who did not return the survey. No further data were available for comparison between those who returned their survey and those who did not.

Sixty-four percent were married (4866/7580), 63% were employed or students (4792/7639) and 37% (2847/7639) were not in employment or were unable to work. The mean age was 50.9 years (SD 14.2).

The mean age of all 17780 patients (60.7% female) sent a screening survey was 46.2 years (SD 15.3). Therefore, patients who completed the screening survey were older and more likely to be female [35].

### Emotional well-being

Overall, 23.9% (1793/7509) scored CES-D ≥ 16 indicating a significant level of depressive symptoms, 21.5% (538/2498) of male and 25.1% (1252/4997) of female patients.

### Hazardous drinking

Almost 15% (752/5061, 14.9%) of females and 28.5% (722/2533) of males reported drinking six or eight standard drinks respectively on one occasion at least monthly; with 6.8% (342/5061) of females and 18% (457/2533) of males reporting doing so at least once a week. Over 16.4% (1245/7602; 95%CI 14.6 to 18.1) met criteria for hazardous drinking in the past 12 months. Fewer females (12.1%, 611/5056) compared to males (25.0%, 633/2532) were hazardous drinkers (OR 0.4; 95%CI 0.4 to 0.5). Almost a third (394/1772, 32.1%) of hazardous drinkers scored CES-D ≥ 16 compared to 22.1% (1378/6230) who were not hazardous drinkers.

### Ever afraid of intimate partner

For individuals who had ever been in an intimate relationship, 16.4% (1213/7372) reported ever being afraid of their partner. A higher proportion of females (1029/4938, 20.8%) compared to males (183/2419, 7.6%) reported ever being afraid of an intimate partner during their lifetime (OR 3.2, 95%CI 2.5 to 4.0). Forty-five percent (536/1203, 44.6%) of participants who had ever been afraid of their partner had depressive symptoms (CES-D ≥ 16), in contrast to 19.5% (1179/6051) of participants that had never been afraid of an intimate partner.

### Psychosocial factors associated with CES-D score

Table 1 shows strong evidence for an association between depressive symptoms and most of the patient factors, except general practice location and patient's country of birth. The results for these patient factors are reported by sex in Table 2. Table 2 shows that men and women who had ever been afraid of a partner, or who were hazardous drinkers, had on average higher depressive symptom scores than those who had never been afraid, or who were not hazardous drinkers.

**Table 1:** Depressive symptoms by patient characteristics and test for interaction for patient characteristics and sex

	N	Mean	(SD)	Diff	(95% CI)	P-value	Interaction term	P-value
**Gender**								
Male	2491	9.76	(10.08)	Ref				
Female	4991	10.9	(10.53)	1.03	(0.51, 1.54)	< 0.001		
**Age group (Years)**								
18-24	323	14.4	(11.44)	Ref				0.18
25-34	842	12.54	(10.82)	-1.84	(-3.15, -0.52)	< 0.001	-3.39	
35-44	1443	11.15	(10.88)	-3.10	(-4.34, -1.86)		-2.88	
45-54	1763	10.83	(10.90)	-3.47	(-4.69, -2.25)		-2.50	
55-64	1694	9.67	(9.95)	-4.59	(-5.81, -3.36)		-3.02	
65-75	1361	8.37	(8.48)	-5.87	(-7.12, -4.61)		-1.68	
**Ever afraid of partner**								
Not ever afraid	6042	9.23	(9.36)	Ref				
Ever afraid of partner	1202	16.49	(12.55)	7.14	(6.52, 7.76)	< 0.001	-2.5	0.003
**Hazardous drinking**								
No	6218	10.01	(10.09)	Ref				
Yes	1228	12.93	(11.42)	2.97	(2.34, 3.60)	< 0.001	1.41	0.03
**Current smoker**								
No	6107	9.55	(9.56)	Ref				
Yes	1357	14.76	(12.58)	5.12	(4.52, 5.72)	< 0.001	-1.1	0.09
**Marital status**								
Never married/single	1334	13.46	(11.63)	Ref		< 0.001		0.01
Widowed/divorced/aaseparated	1312	13.38	(12.28)	-0.11	(-0.88, 0.66)		-1.37	
Married	4770	8.9	(9.04)	-4.51	(-5.14, -3.89)		0.82	
**Sexuality**								
Exclusively heterosexual	6803	10.16	(10.21)	Ref				
Not exclusively heterosexual	354	14.65	(11.76)	4.44	(3.31, 5.57)	< 0.001	2.35	0.05
**Lives alone**								
No	6476	10.11	(10.05)	Ref				
Yes	996	13.17	(12.05)	3.09	(2.40, 3.78)	< 0.001	-3.82	< 0.001
**Employment status**								
Employed/Student	4802	9.93	(9.87)	Ref		< 0.001		0.75
Not employed/not in paid employment	2274	9.89	(9.72)	0.07	(-0.44, 0.57)		-0.06	
Unable to work	401	20.85	(13.85)	11.01	(9.98, 12.04)		0.77	
**Highest level of education**								
Completed year 12 or less	4182	11.07	(10.57)	Ref		< 0.001		0.57
Certificate or diploma	1566	10.53	(10.64)	-0.71	(-1.31, -0.11)		0.65	
Bachelor degree or higher	1718	9.1	(9.52)	-2.18	(-2.79, -1.57)		0.41	
**Pension or benefit main** **source of income**								
No	5518	9.6	(9.60)	Ref				
Yes	1890	13.11	(12.06)	3.62	(3.08, 4.16)	< 0.001	-0.28	0.62
**GP location**								
Urban (RRMA 1 & 2)	5063	10.56	(10.46)	Ref				
Rural (RRMA 3-5)	2433	10.42	(10.22)	-0.14	(-1.22, 0.95)	0.81	-0.55	0.32
**Country of birth**								
Other	1384	10.51	(10.20)	Ref			-0.11	0.86
Australia	6093	10.53	(10.43)	0.11	(-0.50, 0.72)	0.72		
**Long term illness/health** **problem or disability**								
No	4964	8.81	(9.06)	Ref				
Yes	2363	14.06	(12.00)	5.33	(4.83, 5.82)	< 0.001	-1.64	0.002
**Health rating**								
Excellent	763	5.4	(6.79)	Ref		< 0.001		0.05
Very good	2782	7.37	(7.69)	2.04	(1.28, 2.78)		1.53	
Good	2644	11.54	(10.00)	6.18	(5.42, 6.94)		2.07	
Fair	1012	17.19	(12.28)	11.9	(11.02, 12.79)		0.37	
Poor	206	25	(14.18)	19.6	(18.19, 21.08)		1.83	

Ref = reference category for patient characteristics

SD = Standard deviation

Difference in mean depressive symptoms (Diff), respective 95% confidence intervals (CI) and p-values calculated using mixed effects linear regression using restricted maximum likelihood estimation (REML), treating GP practice as a random effect

Interaction term for each patient characteristic and sex and related p-value

**Table 2:** Depressive symptoms by patient characteristics separately for males and females

Males (N = 2491)						
	**n**	**Mean**	**(SD)**	**Diff**	**(95% CI)**	**P-value**

**Ever afraid of partner**						
Not ever afraid	2185	8.88	(9.28)	Ref		
Ever afraid of partner	179	18.23	(12.61)	9.17	(7.66, 10.69)	< 0.001
**Hazardous drinking**						
No	1841	9.09	(9.62)	Ref		
Yes	620	11.52	(11.02)	2.51	(1.58, 3.45)	< 0.001
**Current smoker**						
No	2051	8.73	(8.98)	Ref		
Yes	430	14.73	(13.14)	5.84	(4.78, 6.89)	< 0.001
**Marital status**						
Never married/single	423	13.16	(11.92)			< 0.001
Widowed/divorced/separated	321	14.09	(12.42)	0.89	(-0.59, 2.37)	
Married	1729	8.1	(8.55)	-5.03	(-6.13, -3.94)	
**Sexuality**						
Exclusively heterosexual	2240	9.4	(9.90)	Ref		
Not exclusively heterosexual	147	12.76	(11.12)	3.07	(1.26, 4.88)	0.001
**Live alone**						
No	2171	9.03	(9.41)	Ref		
Yes	314	14.74	(12.76)	5.67	(4.45, 6.89)	< 0.001
**Long term illness/health** **problem or disability**						
No	1405	7.08	(7.52)	Ref		
Yes	1021	13.62	(11.90)	6.57	(5.76, 7.38)	< 0.001
**Health rating**						
Excellent	205	4.9	(6.05)	Ref		< 0.001
Very good	808	5.84	(6.32)	0.94	(-0.49, 2.37)	
Good	962	9.86	(9.17)	4.89	(3.48, 6.30)	
Fair	397	16.58	(12.18)	11.71	(10.13, 13.28)	
Poor	92	23.64	(13.46)	18.66	(16.35, 20.96)	

**Females (N = 4991)**						

	**n**	**Mean**	**(SD)**	**Diff**	**(95% CI)**	**P-value**

**Ever afraid of partner**						
Not ever afraid	3845	9.44	(9.40)	Ref		
Ever afraid of partner	1022	16.19	(12.52)	6.67	(5.99, 7.36)	< 0.001
**Hazardous drinking**						
No	4364	10.41	(10.27)	Ref		
Yes	608	14.38	(11.65)	3.93	(3.05, 4.80)	< 0.001
**Current smoker**						
No	4043	9.98	(9.82)	Ref		
Yes	926	14.78	(12.33)	4.74	(4.01, 5.47)	< 0.001
**Marital status**						
Never married/single	911	13.61	(11.50)			< 0.001
Widowed/divorced/separated	991	13.15	(12.23)	-0.48	(-1.40, 0.43)	
Married	3040	9.36	(9.27)	-4.22	(-4.97, -3.46)	
**Sexuality**						
Exclusively heterosexual	4563	10.53	(10.33)	Ref		
Not exclusively heterosexual	207	15.99	(12.03)	5.41	(3.98, 6.84)	< 0.001
**Live alone**						
No	4304	10.66	(10.32)	Ref		
Yes	682	12.44	(11.65)	1.85	(1.02, 2.68)	< 0.001
**Long term illness/health** **problem or disability**						
No	3552	9.5	(9.53)	Ref		
Yes	1336	14.42	(12.08)	4.93	(4.30, 5.57)	< 0.001
**Health rating**						
Excellent	557	5.58	(7.05)	Ref		< 0.001
Very good	1969	7.99	(8.11)	2.47	(1.59, 3.35)	
Good	1677	12.51	(10.33)	6.96	(6.06, 7.86)	
Fair	612	17.61	(12.36)	12.08	(11.01, 13.15)	
Poor	114	26.1	(14.71)	20.48	(18.60, 22.37)	

Ref = reference category for patient characteristics

SD = Standard deviation

Difference in mean depressive symptoms (Diff), respective 95% confidence intervals (CI) and p-values calculated using mixed effects linear regression using restricted maximum likelihood estimation (REML), treating GP practice as a random effect

Analysis reported separately for males and females when p-value for interaction was 10% or greater in Table 1

### Association between depressive symptoms, hazardous drinking and ever been afraid of partner

Table 3 presents the multivariable model for depressive symptoms where both hazardous drinking and IPV were fitted to the same model for males and females separately. There was stronger association between depressive symptoms and ever being afraid of a partner compared to depressive symptoms and hazardous drinking (Table 3, Model 1). Test for interaction showed no evidence for an interaction between hazardous drinking and ever been afraid of partner for both sexes (interaction term = -2.33, 95%CI: -5.4, 0.75, p-value = 0.14 for males and 0.02, 95%CI: -1.9, 1.9, p-value = 0.98 for females). A small proportion of participants reported positive for hazardous drinking and ever being afraid of intimate partner (2.63% (63/2391) of males and 3.94% (194/4919) of females). The strength of association between depressive symptoms and ever been afraid of a partner, and depressive symptoms and hazardous drinking remained after adjusting for all factors except sexuality (Table 3, model 2).

**Table 3:** Multivariable analysis for Hazardous drinking in last year and ever-afraid of partner

	Males					
	(N = 2336)			(N = 2261)		
	**Model 1**			**Model 2: Adjusted for all factors except sexuality**		

	**Diff**	**(95% CI)**	**P-value**	**Diff**	**(95% CI)**	**P-value**

**Ever afraid of partner**	8.93	(7.47, 10.40)	< 0.001	6.87	(5.42, 8.33)	< 0.001
**Hazardous drinking in last year**	1.99	(1.09, 2.88)	< 0.001	1.07	(0.21, 1.94)	0.015

	**Females**					
	**(N = 4849)**			**(N = 4696)**		

	**Model 1**			**Model 2: Adjusted for all factors except sexuality**		

	**Diff**	**(95% CI)**	**P-value**	**Diff**	**(95% CI)**	**P-value**

**Ever afraid of partner**	6.45	(5.75, 7.15)	< 0.001	5.26	(4.55, 5.97)	< 0.001
**Hazardous drinking in last year**	3.20	(2.33, 4.08)	< 0.001	2.23	(1.35, 3.11)	< 0.001

**Model 1 - Adjusted model - **Ever afraid of partner and hazardous drinking fitted in the same model, but not adjusted for any other

**Model 2 - Adjusted for all factors, except sexuality - **Ever afraid of partner and hazardous drinking fitted in the same model adjusted for all factors: Age group, education level, employment status, pension or benefit as main source of income, marital status, live alone, but not sexuality due to large proportion of missing data

Difference in mean depressive symptoms (Diff), respective 95% confidence intervals (CI) and p-values calculated using mixed effects linear regression using restricted maximum likelihood estimation (REML), treating GP practice as a random effect

## Discussion

### Summary of main findings

Almost a quarter of participants randomly recruited from general practice met criteria for current depressive symptoms (24%). IPV (16%) and hazardous drinking (16%) were equally common among patients in the current study.

Men and women who had ever been afraid of a partner or who were hazardous drinkers had on average higher depressive symptom scores than those who had never been afraid or who were not hazardous drinkers. There was a stronger association between depressive symptoms and ever been afraid of a partner compared to hazardous drinking for both males and females, even after adjusting for age group, income, employment status, marital status, living alone and education level.

IPV and hazardous drinking are serious problems in themselves and are also known to complicate the detection and management of depression in primary care. We do not know whether outcomes for men and women with depression differ for those experiencing one or both of these problems but we are tracking this in the diamond study and will report in due course.

### Comparison with existing literature

A recent Australian report found considerably higher proportions of male (36%) and female (24%) patients drinking heavily than the current study [47]. However, that study had a lower threshold for heavy drinking than the current study (i.e. drinking six or more standard drinks for men (four or more for women) on one occasion at least once a week). Furthermore, it may not be representative of the population who attend general practice in Australia as, unlike the current study; it was biased towards frequent attenders.

Almost a quarter of patients met criteria for depressive symptoms. As expected, these figures are lower than studies where primary care patients are screened in waiting rooms using the CES-D (37% overall (range 24%-55% across countries)). Contributing factors may be that patients in waiting rooms are more likely to be experiencing an acute illness and also that patients with depressive symptoms are high utilizers of primary care services and therefore may be more likely to be recruited into studies that recruit in waiting rooms due to frequent attendance.

Our results on the incidence of potential partner abuse among females are similar to those reported in an Australian study among female patients in general practice waiting rooms [3]. No data are available for comparison among male patients.

### Implications for future research or clinical practice

GPs detect IPV in less than a fifth of female patients experiencing it [48]. GPs play a vital role in the identification and treatment of alcohol problems [24] and it has been argued that they need to play a greater role in IPV, [3] particularly when patients present with depressive symptoms. Duxbury suggests that GPs should enquire about IPV among patients presenting with associated psychological conditions to improve detection [49]. While Feder et al. [50] remind us that not all patients experiencing IPV present with such psychological symptoms,[3] we believe that GPs should enquire about fear of partner and hazardous drinking in patients presenting with depressive symptoms. Strategies to assist primary care doctors to recognise and manage intimate partner violence and hazardous drinking in patients with depression may lead to better outcomes from management of depression in primary care.

We know that GPs can provide effective brief interventions to reduce alcohol consumption,[51] however, there is a lack of evidence around IPV interventions [37,52,53]. We have highlighted the great need for those patients attending with depression to have the opportunity and encouragement to discuss both alcohol use and relationship problems to determine whether associated social or clinical problems require attention as part of the management of depression [54].

### Strengths and limitations of the study

A major strength of this study was the large sample size that enabled testing of interactions between males and females, and separate reporting of results for men and women. This cross-sectional sample has shown the strength of association between hazardous drinking, ever been afraid of their partner and depressive symptoms. The findings presented are limited as causality cannot be implied due to the cross sectional nature of the data. The number of patients with both hazardous drinking and being afraid of partner were small. A small proportion of participants reported positive for hazardous drinking and ever being afraid of intimate partner (63 males and 194 females), so it was not possible to test for the interaction between hazardous drinking and reporting being afraid of partner.

## Conclusions

Being afraid of your partner and hazardous drinking are both associated with depressive symptoms. Yet not everyone who has depressive symptoms will report hazardous drinking or fear of their partner; some will report none, some one and a few, both of these. It is likely that the association with depressive symptoms is bi-directional for both these important problems and our paper highlights the complex relationships with depressive symptoms as they present in primary care. The strong association found in our sample highlights the need to investigate whether interventions designed to assist in recognising and managing these problems results in better outcomes than treating depressive symptoms alone.

## Competing interests

The authors declare that they have no competing interests.

## Authors' contributions

JG conceived of the *diamond *study & all authors contributed to its design. PC analysed & interpreted the data. GG & KH drafted the manuscript and all authors contributed to its revision. All authors approved the final version of the article to be published.

## Pre-publication history

The pre-publication history for this paper can be accessed here:

http://www.biomedcentral.com/1471-2296/11/72/prepub
